# Is Lutikizumab, an Anti–Interleukin-1*α*/*β* Dual Variable Domain Immunoglobulin, efficacious for Osteoarthritis? Results from a bayesian network meta-analysis

**DOI:** 10.1155/2020/9013283

**Published:** 2020-11-04

**Authors:** Ziqin Cao, Yajia Li, Wanchun Wang, Shuo Jie, Xuantao Hu, Jian Zhou, Tong Wu, Dilihumaer Aili, Zeling Long, Yihan Li, Pengcheng Dou, Ren Wu

**Affiliations:** ^1^Department of Orthopedics, The Second Xiangya Hospital, Central South University, Changsha, Hunan 410011, China; ^2^Department of Dermatology, Xiangya Hospital, Central South University, Changsha, China; ^3^Department of Orthopedic Surgery, Mayo Clinic Rochester, USA; ^4^Department of Orthopedic Surgery, University of California, Davis Medical Center, USA

## Abstract

**Objective:**

Most guidelines recommend the use of nonsteroidal anti-inflammatory drugs (NSAIDs), duloxetine, and tramadol for the nonoperative treatment of osteoarthritis (OA), but the use of them is limited by the tolerability and safety concerns. Lutikizumab is a novel anti–IL-1*α*/*β* dual variable domain immunoglobulin that can simultaneously bind and inhibit IL-1*α* and IL-1*β* to relieve the pain and dysfunction symptoms. We conducted this network meta-analysis to comprehensively compare the clinical efficacy and safety of lutikizumab with other drugs recommended by guidelines.

**Methods:**

We conducted a Bayesian network and conventional meta-analyses to compare the efficacy and safety of lutikizumab with other traditional drugs. All eligible randomized clinical trials, in PubMed, CNKI, EMBASE, and Web of Science databases, from January 2000 to January 2020, were included. The Cochrane risk of the bias assessment tool was used for quality assessment. Pain relief, function improvement, and risk of adverse effects (AEs) were compared in this study.

**Results:**

24 articles with 11858 patients were included. Duloxetine (DUL) had the largest effect for pain relief (4.76, 95% CI [2.35 to 7.17]), and selective cox-2 inhibitors (SCI) were the most efficacious treatment for physical function improvement (SMD 3.94, 95% CI [2.48 to 5.40]). Lutikizumab showed no benefit compared with placebo for both pain relief (SMD 1.11, 95% CI [-2.29 to 4.52]) and function improvement (SMD 0.992, 95% CI [-0.433 to 4.25]). Lutikizumab and all other drugs are of favorable tolerance for patients in the treatment of OA compared with placebo.

**Conclusions:**

Lutikizumab, the new anti–Interleukin-1*α*/*β* dual variable domain immunoglobulin, showed no improvement in pain or function when compared with placebo. Selective cox-2 inhibitors and duloxetine remain the most effective and safest treatment for OA. More high-quality trials are still needed to reconfirm the findings of this study.

## 1. Introduction

Osteoarthritis (OA) is the most common form of joint disease, usually affecting load-bearing joints such as hip and knee joints [1]. Approximately 302 million people suffer from OA worldwide every year [2]. OA can lead to local pain and joint stiffness in its early stages and can cause dysfunction and even disability in the late stages. OA-related pain and dysfunction increase the risk of mortality [3] as well as the societal economic burden [4]. To address the health issue, most guidelines recommend the use of nonsteroidal anti-inflammatory drugs (NSAIDs), duloxetine, or tramadol for nonoperative treatment of OA [2]. However, the use of these drugs is limited by tolerability and safety concerns [5].

Previous literature has confirmed that the proinflammatory cytokines, Interleukin-1*α*, and 1*β* (IL-1*α*/*β*) are pain mediators and play an important role in the pathogenesis of OA [6, 7]. Inactive IL-1*α* is stored in the cell or on the cell membrane. Once the cells are damaged, IL-1*α* is activated and released, inducing the activation of IL-1*β*, and finally promoting the progression of OA. [8, 9]. IL-1*α* and IL-1*β* both bind to the IL-1 receptor 1 (IL-1R1), causing joint pain, inflammation, cartilage destruction, and bone resorption [10–13]. In addition, researchers have found that the concentration of IL-1 in the serum and joint fluid of patients with OA is elevated [14, 15]. Subsequently, numerous IL-1R antagonists and IL-1R1 antibodies have been developed. However, clinical trials utilizing them in patients with OA did not report the desired results [16, 17]. Lutikizumab is a new anti–IL-1*α*/*β* dual variable domain immunoglobulin that simultaneously binds and inhibits IL-1*α* and IL-1*β* without interfering with other IL-1 family members such as IL-1Ra [18]. Multiple animal experiments and clinical trials already have shown the potential of lutikizumab for the treatment of OA [19–21].

To comprehensively assess the clinical efficacy, including pain reduction and physical function improvement and the safety of lutikizumab for the treatment of OA, we designed and conducted a Bayesian network meta-analysis. Ten drugs widely used clinically were included in the meta-analysis. Based on these drugs' activity mechanism, we divided them into five groups: anti-Interleukin-1*α*/*β* dual variable domain immunoglobulins (lutikizumab), selective Cox-2 inhibitors (celecoxib and etoricoxib), duloxetine, opioid (tramadol), and traditional NSAIDs (ibuprofen, naproxen, diclofenac, and paracetamol/acetaminophen).

## 2. Method

### 2.1. Literature Search

We conducted a systematic search of the PubMed, CNKI, EMBASE, and Web of Science databases, from January 2000 to January 2020, with the search terms consisted of ((“Lutikizumab” OR “anti-Interleukin-1*α*/*β* dual variable domain immunoglobulin” OR “anti-Interleukin-1*α*/*β*”) OR (“selective cox-2 inhibitor” OR “cox-2 inhibitor” OR “etoricoxib” OR “celecoxib”) OR (“NSAIDs” OR “non-steroidal anti-inflammatory drugs” OR “acetaminophen” OR “diclofenac” OR “naproxen” OR “paracetamol” OR “ibuprofen”) OR (“duloxetine”) OR (“opioids” OR “Tramadol”) AND (“osteoarthritis” OR “degenerative joint disease” OR “OA”)).

Reference lists of relevant systematic reviews and meta-analyses were also reviewed to identify additional eligible studies. Only randomized clinical trials (RCTs) were included, but no restriction was placed on the language of publication

### 2.2. Eligibility Criteria

The inclusion criteria were as follows: (1) Only randomized clinical trials (RCTs) with prospective parallel-group design; (2) Studies comparing the target drugs with each other or placebo in participants with OA at any joint. The exclusion criteria were as follows: (1) Dose-escalation studies of only one drug; (2) Studies on postoperative patients with OA; (3) Reviews, systematic reviews and meta-analyses, case report, conference abstractions, letters, pharmacokinetical or pharmacodynamical studies, and animal experimental studies.

### 2.3. Quality Assessment

Two authors conducted the methodological quality and bias assessment of included studies with the Cochrane risk of the bias assessment tool strictly. The following indexes were evaluated and ranked as low risk of bias, unclear risk of bias, or high risk of bias: sequence generation, allocation concealment, blinding, incomplete outcome data, selection outcome reporting, and other sources of bias [22]. All disputes were resolved through discussion.

### 2.4. Data Extraction

Author, publication year, number of patients, mean age, gender ratio (male/female), diseased joint, funded or not, intervention methods, follow-up period, and outcome data were extracted from included studies. We would give priority to select the data from the intention-to-treat analysis to reduce the withdrawal bias if available. For studies involving multiple treatment groups with different doses of the same drug, we selected the most effective dose group based on the respective study's recommendations [23].

### 2.5. Outcome Measures

The primary efficacy endpoint was pain relief, and the secondary efficacy outcome was function improvement. Considering the differences between the baseline value of each included study, which may lower the reliability of the results and conclusions, the change-from-baseline score at the last follow-up (mean ± SD) was used to evaluate the efficacy to minimize the biases caused by heterogeneity of baseline values. No restriction was placed on the types of questionnaire used in pain evaluation. The function subscales of Western Ontario and McMaster Universities Arthritis Index (WOMAC) were used to evaluate the function improvements preferentially. Any other functional measurement scales, such as the Lequesne index, would be used if no WOMAC function score was reported. Standardised mean difference (SMD) was used because results from different scales were included in the same network [22].

The safety outcomes included the withdrawal due to adverse effects (AEs), serious AEs, and any drug-related AEs. Serious AEs included any AEs that resulted in death, was life-threatening, needed for hospitalization, or prolonged the existing hospitalization, caused disability/incapacity, or caused anomaly/birth defect. The odds ratio (OR) with 95% confidence intervals (CI) was used to measure the safety of target drugs versus placebo or against each other.

### 2.6. Statistical Analysis

Conventional direct meta-analyses comparing the efficacy and safety of treatments with placebo were conducted in Stata/MP (version 14.0, Stata Corp, College Station, Texas, USA). The heterogeneity across studies was tested by the *Q* and *I*^2^ statistic, in which *P* < 0.05 or *I*^2^ > 50% implies significantly heterogeneity. If significant heterogeneity across studies was found, a random-effects model would be used. Otherwise, a fixed-effects model would be preferred.

The random-effects Bayesian network meta-analyses were conducted in Aggregate Data Drug Information System (ADDIS, version 1.16.8). This method can augment the number of studies within each comparison and narrow the CIs' width, and then increase the reliability of result and conclusion [24–27]. Noninformative uniform and normal prior distributions were used in this study, then four different sets of starting values were set to fit the model to yield 40000 iterations (10000 per chain) and obtain the posterior distributions of model parameters [28, 29]. The thinning interval was set at 20 and the burn-ins at 1000 for each chain. Convergence of iterations was assessed using the Gelman-Rubin-Brooks statistic. Consistency of the network meta-analysis was reconfirmed via global inconsistency tests and node-split tests in Stata/MP (version 14.0). SMDs and ORs with 95% CI would be generated from the posterior distribution medians. Significant differences were considered between treatments being compared when the corresponding 95% CI did not contain 0 for the SMD or 1 for OR. Surface under the cumulative ranking (SUCRA) and the cluster-ranking plots were used to rank the efficacy and safety of different treatments. *P* < 0.05 was considered statistically significant.

The following subgroup analyses would be performed if available: according to the drug delivery route (topical, oral, or injective) and according to the diseased joint (hip, knee, hand, or ankle).

## 3. Results

### 3.1. Study Selection

This network meta-analysis was conducted strictly with the Preferred Reporting Items for Systematic Reviews and Meta-Analyses (PRISMA) guidelines [30].

**Figure 1:**
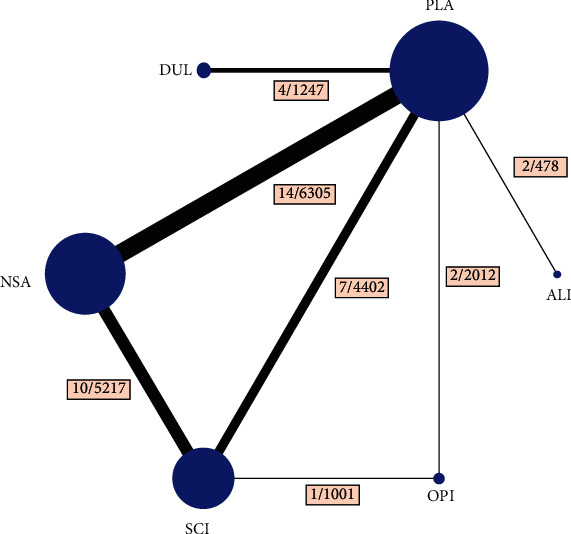
Structure of network formed by interventions. The lines between treatment nodes indicate the direct comparisons made within randomized controlled trials. Numbers (*n*/*n*) near the line indicate “number of trials/number of participants” of the related comparisons.

Twenty-four eligible studies, including 26 trials, were finally identified [31–54]. The details of the selection process are shown in Supplementary Appendix Figure 1. Six treatment arms (anti-Interleukin-1*α*/*β* dual variable domain immunoglobulins (ALI), selective Cox-2 inhibitors (SCI), NSAIDs (NSA), duloxetine (DUL), opioids (OPI), and placebo (PLA)) were included in the network (Figure 1).

### 3.2. Study Characteristics

11858 patients were assessed in this study. Most of the 26 trials included studied knee or hip OA. Only two trials with 541 patients studied hand OA.

Across the trials, the mean age of the patients was 62.35 years (range: 58.15 to 60.00 years). The proportion of male patients was 30.22% (range: 15.31% to 54.03%), and the median follow-up was 84 days (IQR 42–89.25 days). The number of patients enrolled for each treatment was 326 (ALI), 2518 (SCI), 3985 (NSA), 621 (DUL), 1405 (OPI), and 3033 (placebo).

The details of patient baseline characteristics are presented in Supplementary Appendix Table 1. The methodological quality and bias-risk evaluations of all included studies are presented in Supplementary Appendix Table 2. Based on these results, the main contributing factors to risks of bias were performance bias, selection bias, and attrition bias.

### 3.3. Primary Efficacy Endpoint

#### 3.3.1. Conventional Direct Meta-Analysis

Twenty-two trials comparing five drugs with placebo were analyzed. The random-effects model was used because of the heterogeneity of the studies and interventions.

No significant differences were found in the comparison of placebo with ALI (SMD 1.118, 95% CI [-0.374 to 2.610], *P* > 0.05) or OPI (SMD 1.914, 95% CI [-2.833 to 6.660], *P* > 0.05). The other treatments all had greater efficacy than placebo for pain relief. DUL had the greatest efficacy for pain relief (SMD 4.764, 95% CI [3.895 to 5.632], *P* < 0.05). The details of the direct meta-analyses for all treatments compared with placebo are presented in Table 1.

#### 3.3.2. Network Meta-Analysis

Twenty-six trials were analyzed in the pain-relief network. As no significant inconsistency was reported in global inconsistency tests and node-split tests, the consistency model was used.

**Figure 2:**
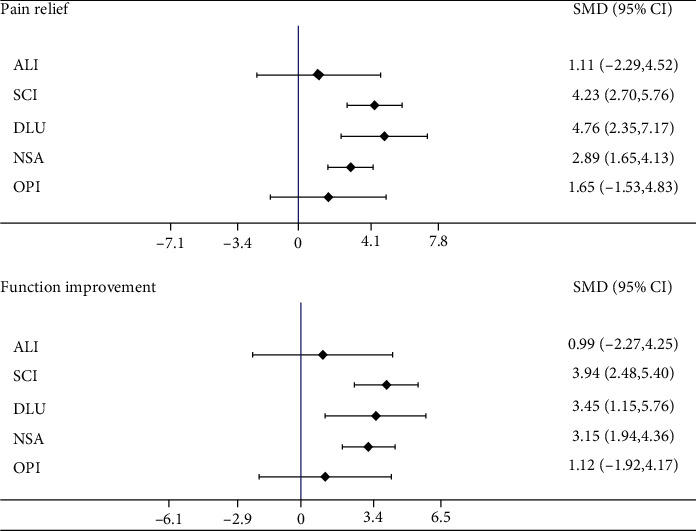
The forest plots. The forest plots of pain relief and function improvement for network meta-analysis. (SMD: standardised mean difference; CI: confidence interval).

DUL was the most efficacious treatment for pain relief (SMD compared with placebo 4.76, 95% CI [2.35 to 7.17]), while both ADL (SMD 1.11, 95% CI [-2.29 to 4.52]) and OPI (SMD 1.65, 95% CI [-1.53 to 4.83]) showed no benefit compared with placebo (Figure 2 and Table 2). According to the SUCRA value, DUL had the greatest effect on pain relief (SUCRA = 88.7%), followed by SCI (SUCRA = 88.4%), and lastly ALI (SUCRA = 28.6%). The detailed results of the SUCRA rank are presented in Supplementary Appendix Table 3.

### 3.4. Secondary Efficacy Endpoint

#### 3.4.1. Conventional Direct Meta-Analysis

Except for ALI (SMD 0.99, 95% CI [-2.27 to 2.417], *P* > 0.05) and OPI (SMD 1.700, 95% CI [-2.920 to 6.320], *P* > 0.05), all other treatments were superior to placebo. SCI had the greatest efficacy for physical function improvement (SMD 4.498, 95% CI [2.402 to 6.594], *P* < 0.05). The details of the direct meta-analyses for all treatments compared with placebo are presented in Table 1.

#### 3.4.2. Network Meta-Analysis

A total of 26 trials were included in the functional improvement network. No significant inconsistency was found, so the consistency model was more suitable statistically than the inconsistency model.

Similar to the results of the direct meta-analysis, nonsignificant differences were found in comparison of placebo with ALI (SMD 0.992, 95% CI [-0.433 to 4.25]) and OPI (SMD 1.12, 95% CI [-1.92 to 4.17]), and SCI was the most efficacious treatment for physical function improvement (SMD 3.94, 95% CI [2.48 to 5.40]) (Figure 2 and Table 2). The results of most SUCRA rankings showed that the most efficacious treatment was SCI (SUCRA = 88.4%), and the least effective one was ALI (SUCRA = 29.6%) (Supplementary Appendix Table 3).

### 3.5. Safety Endpoint

#### 3.5.1. Conventional Direct Meta-Analysis

Twenty trials involving all five therapies were analyzed in the conventional direct meta-analyses. There was no significant heterogeneity reported, and a fixed-effects model was used. DUL, NSA, and OPI had greater rates for all of safety endpoints compared with placebo, while ALI and SCI did not show a significantly higher risk for any safety endpoint. The details of the pairwise meta-analysis for all drugs compared with placebo are shown in Table 1.

#### 3.5.2. Network Meta-Analysis

Twenty-four trials involving all five treatments were analyzed in the safety network. Node-split tests and global inconsistency tests were performed, and no inconsistency was reported. The consistency model was preferred rather than the inconsistency model.

No treatment had more withdrawals due to adverse events (AE), nor a higher incidence of serious AEs, nor any drug-related AEs. Based on the results of the network comparisons, SCI had the lowest rate of withdrawal due to AEs (SURCA 92.6%, OR -0.11, 95% CI [-0.40 to 0.17]), the lowest rate of serious AEs (SURCA 80.2%, OR -0.01, 95% CI [-0.70 to 0.68]), and the lowest rate of drug-related AEs (SURCA 75.5%, OR 0.07, 95% CI [-0.10 to 0.24]). The cluster rank plots showed that SCI was the optimum treatment from the perspective of safety and efficacy (The results of cluster-rank plots can be seen in Supplementary Appendix Figure 2). The relative safety between different treatments is presented in Table 3. The SURCA and relative safety compared to placebo are presented in Supplementary Appendix Table 4.

### 3.6. Subgroup Analysis

Two subgroup analyses were conducted.

The first subgroup analysis conducted exploited the impacts of different drug delivery routes. Three of the 26 trials used topical drug delivery methods. After excluding these studies, no substantial change was revealed. DUL had the largest efficacy for pain relief (SMD 4.76, 95% CI [2.33 to 7.19]), and SCI for functional improvement (SMD 4.19, 95% CI [2.70 to 5.68]). No treatment showed a higher risk of any safety endpoint (Supplementary Appendix Table 5).

In the second subgroup analysis, there were only two trials on hand joints while others all studied knee or hip joints. After excluding these studies, no substantial change was reported. Similarly, DUL had the highest effect for pain relief (SMD 4.76, 95% CI [2.32 to 7.20]), and SCI for functional improvement (SMD 4.07, 95% CI [2.59 to 5.55]). No treatment showed a higher risk of any safety endpoint (Supplementary Appendix Table 5).

## 4. Discussion

This is the first network meta-analysis comparing the efficacy and safety of lutikizumab, the new anti–Interleukin-1*α*/*β* dual variable domain immunoglobulin, for treating OA with drugs recommended by guidelines [2]. We included all available evidence from randomized clinical trials (RCTs) directly or indirectly comparing lutikizumab with traditional treatments for OA and used the Bayesian method to increase the number of comparisons to enhance the power of the study. As mentioned above, considering the difference in the baseline values from different study populations and their influence on the results, we chose the change-from-baseline score as the outcome measure and only included the literature that reported the results of the change-from-baseline score. Our main findings are (1) ALI (lutikizumab) is not associated with pain relief or functional improvement of OA compared with placebo; (2) DUL, SCI, and NSA therapies all can improve every symptom of OA effectively and have a significant advantage over OPI and ALI; (3) SCI, ALI, DUL, NSA, and OPI are tolerated well for patients in long-term treatment of OA compared with placebo. These results indicate that lutikizumab is not suitable for the treatment of OA because it provides no improvement in joint pain and dysfunction, while selective Cox-2 inhibitors (such as celecoxib and etoricoxib) are the ideal choice for the treatment of OA from the perspective of safety and efficacy. Combined with the results from other clinical trials [16, 17], the inhibition of IL-*α*/*β* does not seem to be a new way to treat OA in the future.

There are several limitations in this study. Considering the unmanageable confounding factors in non-RCTs and their unpredictable influences on the results of network meta-analysis, only RCTs were included. Nevertheless, non-RCTs, especially observational studies, can provide valuable insight into the long-term effectiveness and safety of treatment for OA. To enhance the credibility of this meta-analysis, only high-quality studies were included. This may have contributed to the small number of studies included. Publication bias could be a significant problem for this study, especially the funnel plots that showed a dubious asymmetry. We tried to adjust the publication bias using the trimming and filling method. However, a previous study suggested that the results of the trimming and filling method should be interpreted as a sensitivity analysis rather than a corrected estimate of publication bias [55]. So, the results of this study should be interpreted cautiously, particularly for ALI in which the number of included studies is smaller compared with other treatments. Although we have conducted two subgroup analyses to reduce the impact of potential confounding factors, there are still many other factors that could affect the reliability of the results, such as the differences in comorbidities, duration of OA, and grade of OA in the study populations. For instance, comorbidities usually cause worse symptom management and consequentially affect the results of analgesic effectiveness assessment. Paradoxically, research on analgesics often excludes people with clinically significant comorbidities and does not systematically describe the distribution of comorbidities in the study population. Most of the included studies, coincidentally, failed to report an accurate grade or duration of OA. We were unable to adjust for these factors because of the insufficiency of the related data, and thus those results should be interpreted with caution. More high-quality trials are needed.

## 5. Conclusion

24 studies, involving 26 trials assessing 11858 patients, were included in this network meta-analysis. The results show that lutikizumab, the new anti–Interleukin-1*α*/*β* dual variable domain immunoglobulin, did not improve pain or function in the comparison with placebo. Selective cox-2 inhibitors and duloxetine remain the most effective and safest treatment for OA. More high-quality trials are needed to reconfirm the findings of this study.

## Figures and Tables

**Table 1:** Characteristics of the included comparisons and the results of direct pair-wise meta-analysis (No. of pts: number of patients included; No. of trials: number of trials included into direct pair-wise meta-analysis; SMD: standardised mean difference; OR: odds ratio).

Comparison	No. of trials	No. of pts	Target joint	SMD (95% CI) for pain relief	SMD (95% CI) for function improvement	OR (95% CI) for withdrawal due to AEs	OR (95% CI) for serious AEs	OR (95% CI) for any drug-related AEs
ALI vs. PLA	2	478	Hand, hip, and knee	1.118 (-0.374 to 2.610)	0.992 (-0.433 to 2.417)	1.408 (0.647 to 3.061)	0.595 (0.211 to 1.681)	1.014 (0.732 to 1.405)
SCI vs. PLA	7	4402	Hip and knee	4.594 (2.381 to 6.807)	4.498 (2.402 to 6.594)	0.899 (0.631 to 1.282)	0.947 (0.329 to 2.727)	1.062 (0.890 to 1.267)
DUL vs. PLA	2	1247	Hip and knee	4.764 (3.895 to 5.632)	3.455 (2.470 to 4.440)	2.766 (1.721 to 4.445)	1.018 (0.309 to 3.352)	1.337 (1.098 to 1.627)
NSA vs. PLA	14	6305	Hand, hip and knee	2.688 (1.741 to 3.635)	2.849 (1.819 to 3.878)	1.473 (1.119 to 1.940)	1.758 (0.987 to 3.133)	1.332 (1.129 to 1.572)
OPI vs. PLA	2	2012	Hip and knee	1.914 (-2.833 to 6.660)	1.700 (-2.920 to 6.320)	2.419 (1.600 to 3.656)	1.012 (0.063 to 16.241)	1.281 (1.028 to 1.596)

**Table 2:** Detailed results of network meta-analysis for pain (Bold) and function (Italic) (Data are standardised mean difference, from the top left to the bottom right, higher comparator vs. lower comparator, and their related 95% CI).

ALI	*2.95 (-0.62 to 6.53)*	*2.46 (-1.53 to 6.45)*	*2.16 (-1.31 to 5.64)*	*0.14 (-4.32 to 4.60)*	*-0.99 (-4.25 to 2.27)*
**-3.11 (-6.85 to 0.62)**	SCI	*-0.49 (-3.22 to 2.24)*	*-0.79 (-2.18 to 0.59)*	*-2.82 (-6.01 to 0.37)*	*-3.94 (-5.40 to -2.48)*
**-3.65 (-7.82 to 0.53)**	**-0.53 (-3.39 to 2.33)**	DLU	*-0.30 (-2.91 to 2.30)*	*-2.33 (-6.14 to 1.49)*	*-3.45 (-5.76 to -1.15)*
**-1.77 (-5.40 to 1.85)**	**1.34 (-0.07 to 2.76)**	**1.87 (-0.84 to 4.59)**	NSA	*-2.02 (-5.23 to 1.18)*	*-3.15 (-4.36 to -1.94)*
**-0.54 (-5.20 to 4.13)**	**2.58 (-0.76 to 5.91)**	**3.11 (-0.89 to 7.10)**	**1.23 (-2.10 to 4.57)**	OPI	*-1.12 (-4.17 to 1.92)*
**1.11 (-2.29 to 4.52)**	**4.23 (2.70 to 5.76)**	**4.76 (2.35 to 7.17)**	**2.89 (1.65 to 4.13)**	**1.65 (-1.53 to 4.83)**	PLA

**Table 3:** Detailed results of network meta-analysis for safety endpoints (Data are odds ratio, from the top left to the bottom right, higher comparator vs. lower comparator, and their related 95% CI).

AEs						
	ALI	-0.47 (-1.31 to 0.37)	0.72 (-0.20 to 1.65)	0.02 (-0.81 to 0.85)	0.59 (-0.28 to 1.47)	-0.36 (-1.15 to 0.43)
	0.47 (-0.37 to 1.31)	SCI	1.19 (0.63 to 1.75)	0.49 (0.28 to 0.70)	1.06 (0.65 to 1.48)	0.11 (-0.17 to 0.40)
	-0.72 (-1.65 to 0.20)	-1.19 (-1.75 to -0.63)	Dul	-0.70 (-1.25 to -0.15)	-0.13 (-0.74 to 0.48)	-1.08 (-1.56 to -0.60)
	-0.02 (-0.85 to 0.81)	-0.49 (-0.70 to -0.28)	0.70 (0.15 to 1.25)	NSA	0.57 (0.15 to 0.99)	-0.38 (-0.64 to -0.12)
	-0.59 (-1.47 to 0.28)	-1.06 (-1.48 to -0.65)	0.13 (-0.48 to 0.74)	-0.57 (-0.99 to -0.15)	OPI	-0.95 (-1.33 to -0.57)
	0.36 (-0.43 to 1.15)	-0.11 (-0.40 to 0.17)	1.08 (0.60 to 1.56)	0.38 (0.12 to 0.64)	0.95 (0.57 to 1.33)	Placebo
Serious AEs						
	ALI	0.54 (-0.72 to 1.80)	0.58 (-1.03 to 2.19)	1.03 (-0.18 to 2.24)	0.56 (-2.22 to 3.34)	0.55 (-0.50 to 1.60)
	-0.54 (-1.80 to 0.72)	SCI	0.04 (-1.36 to 1.44)	0.49 (0.10 to 0.88)	0.02 (-2.59 to 2.64)	0.01 (-0.68 to 0.70)
	-0.58 (-2.19 to 1.03)	-0.04 (-1.44 to 1.36)	Dul	0.45 (-0.91 to 1.81)	-0.02 (-2.87 to 2.84)	-0.03 (-1.25 to 1.19)
	-1.03 (-2.24 to 0.18)	-0.49 (-0.88 to -0.10)	-0.45 (-1.81 to 0.91)	NSA	-0.47 (-3.08 to 2.14)	-0.48 (-1.08 to 0.12)
	-0.56 (-3.34 to 2.22)	-0.02 (-2.64 to 2.59)	0.02 (-2.84 to 2.87)	0.47 (-2.14 to 3.08)	OPI	-0.01 (-2.59 to 2.57)
	-0.55 (-1.60 to 0.50)	-0.01 (-0.70 to 0.68)	0.03 (-1.19 to 1.25)	0.48 (-0.12 to 1.08)	0.01 (-2.57 to 2.59)	PLA
Any drug-related AEs						
	ALI	-0.08 (-0.88 to 0.72)	0.44 (-0.38 to 1.25)	0.20 (-0.60 to 1.00)	0.59 (-0.24 to 1.42)	-0.15 (-0.93 to 0.63)
	0.08 (-0.72 to 0.88)	SCI	0.51 (0.23 to 0.80)	0.28 (0.15 to 0.40)	0.67 (0.36 to 0.98)	-0.07 (-0.24 to 0.10)
	-0.44 (-1.25 to 0.38)	-0.51 (-0.80 to -0.23)	Dul	-0.24 (-0.52 to 0.05)	0.15 (-0.21 to 0.52)	-0.59 (-0.82 to -0.36)
	-0.20 (-1.00 to 0.60)	-0.28 (-0.40 to -0.15)	0.24 (-0.05 to 0.52)	NSA	0.39 (0.08 to 0.70)	-0.35 (-0.51 to -0.19)
	-0.59 (-1.42 to 0.24)	-0.67 (-0.98 to -0.36)	-0.15 (-0.52 to 0.21)	-0.39 (-0.70 to -0.08)	OPI	-0.74 (-1.03 to -0.46)
	0.15 (-0.63 to 0.93)	0.07 (-0.10 to 0.24)	0.59 (0.36 to 0.82)	0.35 (0.19 to 0.51)	0.74 (0.46 to 1.03)	Placebo

## Data Availability

Data generated or analyzed during this study are included in this published article. The datasets generated during and/or analyzed during the current study are available from the corresponding author on reasonable request.

## Supplementary Materials

Supplementary Materials The Supplementary Material includes 5 supplementary appendix tables and 2 supplementary appendix figures which support our findings.
