# Supplementary material for: Is Lutikizumab, an Anti–Interleukin-1α/β Dual Variable Domain Immunoglobulin, efficacious for Osteoarthritis? Results from a bayesian network meta-analysis
Source: Biomed Res Int. 2020 Nov 4;2020:9013283. doi: 10.1155/2020/9013283 (PMC7661137; doi:10.1155/2020/9013283)
Supplement: Supplementary Materials — The Supplementary Material includes 5 supplementary appendix tables and 2 supplementary appendix figures which support our findings. [file 9013283.f1.zip › 9013283.f2.pdf]

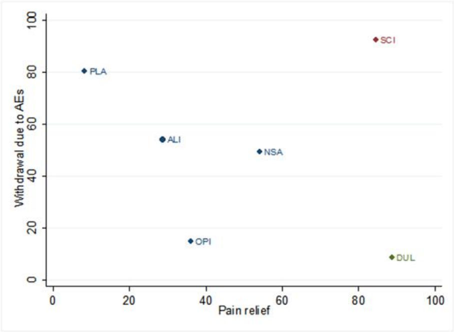

A

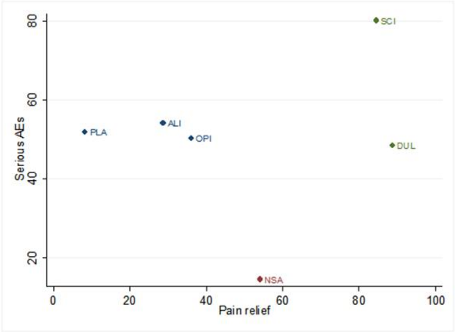

B

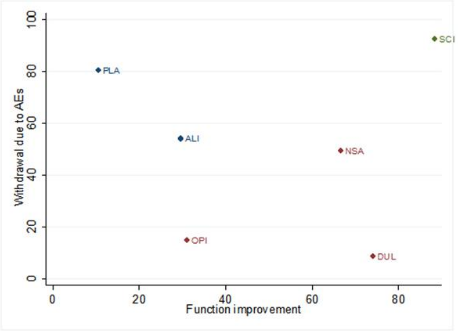

D

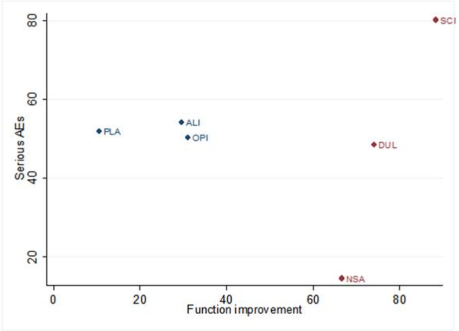

E

Supplementary appendix Figure 2. Cluset-rank plots. (A) The cluster-rank plot of pain relief and withdrawal due to AEs for different treatments. (B) The cluster-rank plot of pain relief and serious AEs for different treatments. (C) The cluster-rank plot of pain relief and drug-related AEs for different treatments. (D) The cluster-rank plot of function improvement and withdrawal due to AEs for different treatments. (E) The cluster-rank plot of function improvement and serious AEs for different treatments. (F) The cluster-rank plot of function improvement and drug-related AEs for different treatments. (The cluster-rank value is the product of the abscissa and ordinate of each treatment).
