# Supplementary material for: Is Lutikizumab, an Anti–Interleukin-1α/β Dual Variable Domain Immunoglobulin, efficacious for Osteoarthritis? Results from a bayesian network meta-analysis
Source: Biomed Res Int. 2020 Nov 4;2020:9013283. doi: 10.1155/2020/9013283 (PMC7661137; doi:10.1155/2020/9013283)
Supplement: Supplementary Materials — The Supplementary Material includes 5 supplementary appendix tables and 2 supplementary appendix figures which support our findings. [file 9013283.f1.zip › 9013283.f3.pdf]

Supplementary appendix Table 1. Baseline Characteristics of included Studies

Author	No.	Year	Number of patients	Mean age	Gender ratio (Male/Female)	Drug delivery route	Diseased joint	Funded or Sponsored	Experimental intervention	Control intervention I	Control intervention II	Follow-up period, days
Zacher J et al [31].	1	2003	516	63.05	101/415	Oral	Hip and Knee	Yes	Etoricoxib	Diclofenac	/	42
Puopolo A et al [32].	2	2007	548	62.56	133/415	Oral	Hip and Knee	No	Placebo	Etoricoxib	Ibuprofen	84
Prior MJ et al [33].	3	2014	542	61.70	139/403	Oral	Hip and Knee	No	Acetaminophen	Placebo	/	84
Emery P et al [34].	4	2008	248	64.02	134/114	Oral	Hip	Yes	Celecoxib	Diclofenac	/	84
McKenna F et al [35].	5	2001	600	61.67	208/392	Oral	Knee	Yes	Placebo	Celecoxib	Diclofenac	42
Altman RD et al [36].	6	2015	385	64.13	89/296	Topical	Hand	No	Diclofenac	Placebo	/	56
Bookman AA et al [37].	7	2004	248	61.80	91/157	Topical	Knee	No	Diclofenac	Vehicle	Placebo	28
Gibofsky A et al [38].	8	2014	305	61.63	102/203	Oral	Hip and Knee	Yes	Diclofenac	Placebo	/	84
Essex MN et al [39].	9	2016	367	64.77	120/247	Oral	Knee	Yes	Celecoxib	Naproxen	Placebo	42
Reed K et al [40].	10	2018	676	60.77	251/425	Oral	Hip and Knee	Yes	Paracetamol	Placebo	/	84

Essex MN et al [41].	11	2012	589	60.38	199/390	Oral	Knee	Yes	Celecoxib	Naproxen	/	180
Leung AT et al [42].	12	2002	501	63.16	109/392	Oral	Hip and Knee	Yes	Placebo	Etoricoxib	Naproxen	84
Gordo AC et al [43].	13	2016	388	62.95	106/282	Oral	Knee	Yes	Celecoxib	Ibuprofen	Placebo	42
Niethard FU et al [44].	14	2005	237	66.00	87/150	Topical	Knee	No	Diclofenac	Placebo	/	21
DeLemos BP et al [45].	15	2011	1001	60.02	369/632	Oral	Hip and Knee	Yes	Tramadol	Celecoxib	Placebo	84
Reginster JY et al [46].	16	2006	997	62.77	279/718	Oral	Hip and Knee	No	Placebo	Etoricoxib	Naproxen	84
Reginster JY et al [46].	17	2006	463	65.70	116/347	Oral	Hip and Knee	No	Etoricoxib	Naproxen	/	280
Gana TJ et al [47].	18	2006	1011	58.15	380/631	Oral	Hip and Knee	Yes	Placebo	Tramadol	/	84
Svensson O et al [48].	19	2006	156	58.66	49/107	Oral	Hip	Yes	Naproxen	Placebo	/	42
Svensson O et al [48].	20	2006	355	60.11	84/271	Oral	Knee	Yes	Naproxen	Placebo	/	42
Fleischmann RM et al [49].	21	2019	347	60.11	122/225	Injective	Knee	Yes	Placebo	Lutikizumab	/	364
Kloppenburg M et al [50].	22	2018	131	66	20/111	Injective	Hand	Yes	Placebo	Lutikizumab	/	182

Chappell AS et al [51].	23	2011	256	62.55	61/195	Oral	Knee	Yes	Placebo	Duloxetine	/	91
Uchio Y et al [52].	24	2018	353	65.58	79/264	Oral	Knee	Yes	Placebo	Duloxetine	/	126
Chappell AS et al [53].	25	2009	231	62.31	60/151	Oral	Knee	Yes	Duloxetine	Placebo	/	84
Wang G et al [54].	26	2016	407	60.51	96/311	Oral	Hip and Knee	Yes	Placebo	Duloxetine	/	91
