# Supplementary material for: Is Lutikizumab, an Anti–Interleukin-1α/β Dual Variable Domain Immunoglobulin, efficacious for Osteoarthritis? Results from a bayesian network meta-analysis
Source: Biomed Res Int. 2020 Nov 4;2020:9013283. doi: 10.1155/2020/9013283 (PMC7661137; doi:10.1155/2020/9013283)
Supplement: Supplementary Materials — The Supplementary Material includes 5 supplementary appendix tables and 2 supplementary appendix figures which support our findings. [file 9013283.f1.zip › 9013283.f4.pdf]

Supplementary appendix Table 2. Methodological quality and risk of bias evaluation.

Author	1.Sequence generation	2.Allocation concealment	3.Blinding	4.Incomplete outcome data	5.Selective outcome reporting	6.Other source of bias
Zacher J et al [31].	Low risk of bias	Low risk of bias	Low risk of bias	Low risk of bias	Low risk of bias	Unclear risk of bias
Puopolo A et al [32].	Low risk of bias	Low risk of bias	Low risk of bias	Low risk of bias	Low risk of bias	Unclear risk of bias
Prior MJ et al [33].	Low risk of bias	Low risk of bias	Low risk of bias	Low risk of bias	Low risk of bias	Unclear risk of bias
Emery P et al [34].	Low risk of bias	Low risk of bias	Low risk of bias	Low risk of bias	Low risk of bias	Unclear risk of bias
McKenna F et al [35].	Low risk of bias	Low risk of bias	Low risk of bias	Low risk of bias	Low risk of bias	Unclear risk of bias
Altman RD et al [36].	Unclear risk of bias	Low risk of bias	Low risk of bias	Low risk of bias	Low risk of bias	Unclear risk of bias
Bookman AA et al [37].	Unclear risk of bias	Low risk of bias	Low risk of bias	Low risk of bias	Low risk of bias	Unclear risk of bias
Gibofsky A et al [38].	Unclear risk of bias	Low risk of bias	Low risk of bias	Low risk of bias	Low risk of bias	Unclear risk of bias

Essex MN et al [39].	Unclear risk of bias	Low risk of bias	Low risk of bias	Low risk of bias	Low risk of bias	Unclear risk of bias
Reed K et al [40].	Low risk of bias	Low risk of bias	Low risk of bias	Low risk of bias	Low risk of bias	Unclear risk of bias
Essex MN et al [41].	Low risk of bias	Low risk of bias	Low risk of bias	Low risk of bias	Low risk of bias	Unclear risk of bias
Leung AT et al [42].	Low risk of bias	Low risk of bias	Low risk of bias	Low risk of bias	Low risk of bias	Unclear risk of bias
Gordo AC et al [43].	Low risk of bias	Low risk of bias	Low risk of bias	Low risk of bias	Low risk of bias	Unclear risk of bias
Niethard FU et al [44].	Low risk of bias	Low risk of bias	Low risk of bias	Low risk of bias	Low risk of bias	High risk of bias
DeLemos BP et al [45].	Unclear risk of bias	Low risk of bias	Low risk of bias	Low risk of bias	Low risk of bias	Low risk of bias
Reginster JY et al [46].	Low risk of bias	Low risk of bias	Low risk of bias	Low risk of bias	Low risk of bias	Low risk of bias
Reginster JY et al [46].	Low risk of bias	Low risk of bias	Low risk of bias	Low risk of bias	Low risk of bias	Low risk of bias

Gana TJ et al [47].	Low risk of bias	Low risk of bias	Low risk of bias	Low risk of bias	Low risk of bias	Low risk of bias
Svensson O et al [48].	Unclear risk of bias	Unclear risk of bias	Low risk of bias	Low risk of bias	Low risk of bias	Low risk of bias
Svensson O et al [48].	Unclear risk of bias	Unclear risk of bias	Low risk of bias	Low risk of bias	Low risk of bias	Low risk of bias
Fleischmann RM et al [49].	Unclear risk of bias	Unclear risk of bias	Low risk of bias	Low risk of bias	Low risk of bias	Unclear risk of bias
Kloppenburg M et al [50].	Unclear risk of bias	Unclear risk of bias	Low risk of bias	Low risk of bias	Low risk of bias	Unclear risk of bias
Chappell AS et al [51].	Low risk of bias	Unclear risk of bias	Low risk of bias	Low risk of bias	Low risk of bias	Unclear risk of bias
Uchio Y et al [52].	Low risk of bias	Unclear risk of bias	Low risk of bias	Low risk of bias	Low risk of bias	Unclear risk of bias
Chappell AS et al [53].	Low risk of bias	Low risk of bias	Low risk of bias	Low risk of bias	Low risk of bias	Unclear risk of bias
Wang G et al [54].	Low risk of bias	High risk of bias	Low risk of bias	Low risk of bias	Low risk of bias	Unclear risk of bias
