# Supplementary material for: Is Lutikizumab, an Anti–Interleukin-1α/β Dual Variable Domain Immunoglobulin, efficacious for Osteoarthritis? Results from a bayesian network meta-analysis
Source: Biomed Res Int. 2020 Nov 4;2020:9013283. doi: 10.1155/2020/9013283 (PMC7661137; doi:10.1155/2020/9013283)
Supplement: Supplementary Materials — The Supplementary Material includes 5 supplementary appendix tables and 2 supplementary appendix figures which support our findings. [file 9013283.f1.zip › 9013283.f5.pdf]

**Supplementary appendix Table 3.** The details of SURCA and mean rank of efficacy endpoints.

| Treatment | SMD (95%CI) for pain relief | SURCA for pain relief, % | Mean rank for pain relief | SMD (95%CI) for Function imrovement | SURCA for Function imrovement, % | Mean rank for Function imrovement |
|---|---|---|---|---|---|---|
| Placebo | Reference | 8.2 | 5.6 | Reference | 10.4 | 5.5 |
| ALI | 1.11 (-2.29 to 4.52) | 28.6 | 4.6 | 0.99 (-2.27 to 4.25) | 29.6 | 4.5 |
| SCI | 4.23 (2.70 to 5.76) | 84.4 | 1.8 | 3.94 (2.48 to 5.40) | 88.4 | 1.6 |
| DUL | 4.76 (2.35 to 7.17) | 88.7 | 1.6 | 3.45 (1.15 to 5.76) | 74 | 2.3 |
| NSA | 2.89 (1.65 to 4.13) | 54.1 | 3.3 | 3.15 (1.94 to 4.36) | 66.6 | 2.7 |
| OPI | 1.65 (-1.53 to 4.83) | 36.0 | 4.2 | 1.12 (-1.92 to 4.17) | 31 | 4.4 |
