# Supplementary material for: Is Lutikizumab, an Anti–Interleukin-1α/β Dual Variable Domain Immunoglobulin, efficacious for Osteoarthritis? Results from a bayesian network meta-analysis
Source: Biomed Res Int. 2020 Nov 4;2020:9013283. doi: 10.1155/2020/9013283 (PMC7661137; doi:10.1155/2020/9013283)
Supplement: Supplementary Materials — The Supplementary Material includes 5 supplementary appendix tables and 2 supplementary appendix figures which support our findings. [file 9013283.f1.zip › 9013283.f6.pdf]

Supplementary appendix Table 4. The details of SURCA and mean rank of safety endpoints.

Treatment	OR (95%CI) for withdrawal due to AEs	SURCA for withdrawal due to AEs, %	Mean rank for withdrawal due to AEs	OR (95%CI) for serious AEs	SURCA for serious AEs, %	Mean rank for serious AEs	OR (95%CI) for any drug-related AEs	SURCA for any drug-related AEs, %	Mean rank for any drug-related AEs
Placebo	Reference	80.6	2	Reference	52.0	3.4	Reference	89.0	1.6
ALI	0.36 (-0.43 to 1.15)	54.1	3.3	-0.55 (-1.60 to 0.50)	54.2	3.3	0.15 (-0.63 to 0.93)	64.6	2.8
SCI	-0.11 (-0.40 to 0.17)	92.6	1.4	-0.01 (-0.70 to 0.68)	80.2	2.0	0.07 (-0.10 to 0.24)	75.5	2.2
DUL	1.08 (0.60 to 1.56)	8.5	5.6	0.03 (-1.19 to 1.25)	48.6	3.6	0.59 (0.36 to 0.82)	19.7	5.0
NSA	0.38 (0.12 to 0.64)	49.4	3.5	0.48 (-0.12 to 1.08)	14.6	5.3	0.35 (0.19 to 0.51)	45.1	3.7
OPI	0.95 (0.57 to 1.33)	14.8	5.3	0.01 (-2.57 to 2.59)	50.4	3.5	0.74 (0.46 to 1.03)	6.2	5.7
