# Supplementary material for: Is Lutikizumab, an Anti–Interleukin-1α/β Dual Variable Domain Immunoglobulin, efficacious for Osteoarthritis? Results from a bayesian network meta-analysis
Source: Biomed Res Int. 2020 Nov 4;2020:9013283. doi: 10.1155/2020/9013283 (PMC7661137; doi:10.1155/2020/9013283)
Supplement: Supplementary Materials — The Supplementary Material includes 5 supplementary appendix tables and 2 supplementary appendix figures which support our findings. [file 9013283.f1.zip › 9013283.f7.pdf]

Supplementary appendix Table 5. The details of results and SURCA for subgroup analyses.

Subgroup analysis	Treatment	SMD (95% CI) for pain relief	SURCA for pain relief , %	SMD (95% CI) for function improvement	SURCA for function improvement, %	OR (95% CI) for withdral due to AEs	SURCA for withdrawal due to AEs, %	OR (95% CI) for serious AEs or death	SURCA for Serious AEs or death, %	OR (95% CI) for any drug-related AEs	SURCA for any drug-related AEs, %
Subgroup of different drug delivery route											
	PLA	Reference	8.1	Reference	10.1	Reference	79.6	Reference	56.2	Reference	86.9
	ALI	1.11 (-2.32 to 4.55)	27.2	0.99 (-2.24 to 4.21)	28.2	0.36 (-0.43 to 1.15)	53.6	-0.55 (-1.60 to 0.50)	81.9	0.15 (-0.63 to 0.93)	79.3
	SCI	4.48 (2.89 to 6.06)	85.8	4.19 (2.70 to 5.68)	88.9	-0.14 (-0.44 to 0.15)	93.5	0.14 (-0.62 to 0.89)	47.0	0.06 (-0.11 to 0.23)	73.8
	DLU	4.76 (2.33 to 7.19)	85.7	3.45 (1.17 to 5.73)	70.1	1.08 (0.60 to 1.56)	6.5	0.03 (-1.19 to 1.25)	51.7	0.59 (0.36 to 0.82)	10.7
	NSA	3.29 (1.90 to 4.69)	58.0	3.57 (2.23 to 4.92)	72.0	0.34 (0.07 to 0.61)	50.0	0.63 (-0.05 to 1.32)	12.3	0.34 (0.17 to 0.50)	31.5
	OPI	1.72 (-1.49 to 4.93)	35.3	1.19 (-1.82 to 4.21)	30.7	0.94 (0.56 to 1.32)	16.8	0.05 (-2.53 to 2.63)	50.9	0.74 (0.46 to 1.02)	17.8

Subgroup of different diseased joint											
	PLA	Reference	7.7	Reference	9.2	Reference	76.4	Reference	53.4	Reference	85.2
	ALI	1.87 (-3.00 to 6.74)	38.7	1.71 (-2.91 to 6.34)	40.1	0.17 (-0.73 to 1.07)	63.6	-0.78 (-2.02 to 0.46)	85.3	0.04 (-1.06 to 1.13)	68.1
	SCI	4.35 (2.79 to 5.90)	82.4	4.07 (2.59 to 5.55)	86	-0.13 (-0.42 to 0.16)	91	0.07 (-0.68 to 0.81)	50.2	0.05 (-0.12 to 0.22)	75.0
	DLU	4.76 (2.32 to 7.20)	85.4	3.45 (1.14 to 5.76)	69.5	1.08 (0.60 to 1.56)	7.3	0.03 (-1.19 to 1.25)	48.9	0.59 (0.36 to 0.82)	20.1
	NSA	3.08 (1.78 to 4.37)	52.4	3.36 (2.10 to 4.63)	66	0.35 (0.09 to 0.62)	47	0.56 (-0.11 to 1.23)	13.1	0.33 (0.16 to 0.49)	45.3
	OPI	1.69 (-1.53 to 4.90)	33.4	1.16 (-1.90 to 4.22)	29.2	0.94 (0.57 to 1.32)	14.7	0.03 (-2.55 to 2.61)	49.0	0.74 (0.45 to 1.02)	4.2
