# Supplementary figures and images for: Is Lutikizumab, an Anti–Interleukin-1α/β Dual Variable Domain Immunoglobulin, efficacious for Osteoarthritis? Results from a bayesian network meta-analysis
Source: Biomed Res Int. 2020 Nov 4;2020:9013283. doi: 10.1155/2020/9013283 (PMC7661137; doi:10.1155/2020/9013283)

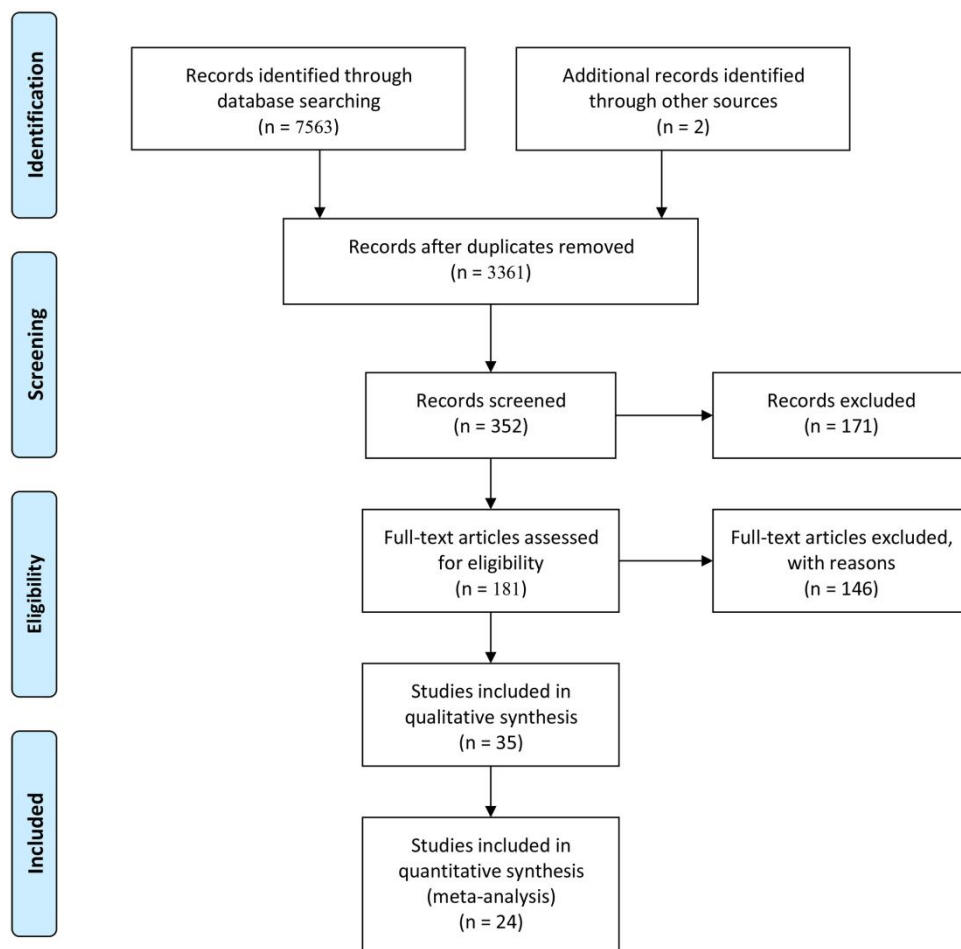

**Supplementary appendix Figure 1. PRISMA Flow Diagram.**

Supplement: Supplementary Materials — The Supplementary Material includes 5 supplementary appendix tables and 2 supplementary appendix figures which support our findings. [file 9013283.f1.zip › 9013283.f1.pdf]
